# The Annoying Act of Creating a Fenestration∗

**DOI:** 10.1016/j.jacadv.2024.100844

**Published:** 2024-02-02

**Authors:** Yves d'Udekem, Alyssia Venna

**Affiliations:** Division of Cardiovascular Surgery, Children’s National Hospital, Washington, DC, USA

**Keywords:** fenestration, Fontan, pulmonary artery pressure

**Figure:**
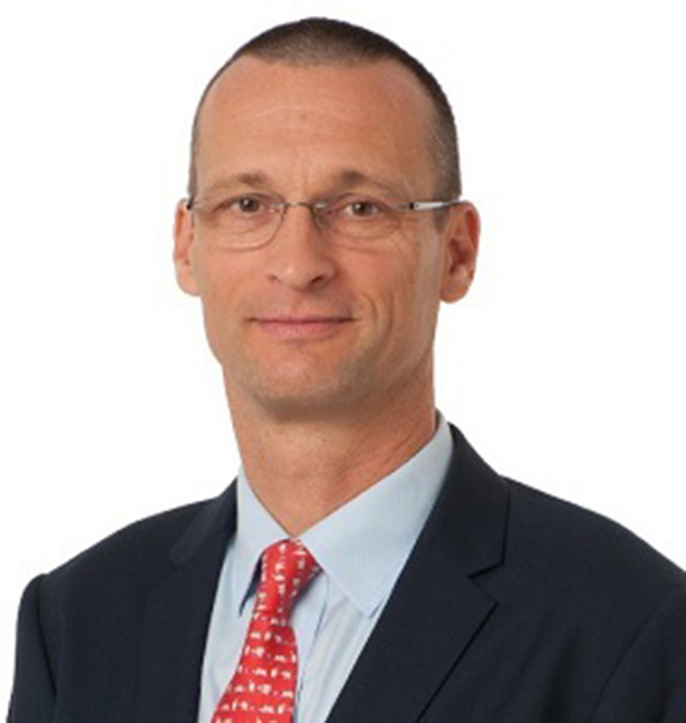

The manuscript of Hill et al^1^ is a welcomed addition to what seems like an endless debate about whether a fenestration should be placed at the time of Fontan completion. With a very large dataset emanating from 7 centers contributing data to the Pediatric Heart Network, they were able to identify that patients will have a shorter hospital stay at the time of the Fontan if a fenestration is present and that the highest difference in hospital stay was found in patients who had preoperative pulmonary artery pressures superior to 13 mm Hg. What can we infer from this work for our practice?

But first, there is 1 detail in this work that may go unnoticed and that we found interesting. Let us look at the data from a surgeon’s perspective. We have had lengthy discussions about the rationale of creating a policy in favor of leaving a fenestration at the time of Fontan completion because supposedly, not all patients need a fenestration, and it might be beneficial for some to not have one. It is estimated that 74% of Fontan procedures performed around the world are extracardiac conduits.^2^ In the current work by Hill et al,^1^ a larger proportion of 28% of the operations studied were lateral tunnel, and this is largely because the work includes 2 centers that almost exclusively use the lateral tunnel and one that has often favored it. Close to half of the patients undergoing an extra-cardiac conduit had a fenestration. This proportion is opposed to 96% of those with a lateral tunnel Fontan having a fenestration. And apart from the type of Fontan, the authors cannot find any patient characteristics that can predict who will receive a fenestration. So, are the surgeons who are doing the lateral tunnel all universally convinced of the benefits of the fenestration while, extraordinarily, only half of the surgeons doing the extra-cardiac conduits are? Of course not. The only reason why all surgeons doing lateral tunnel are leaving a fenestration is because it is so easy to perform that procedure. A quick punch hole is all it takes. On the contrary, when one must do a fenestration in an extra-cardiac conduit, this surgeon has to do a difficult clamping of the atrial tissue to perform the fenestration on the beating heart. This can be rather annoying because you are working with tiny bits of tissue cramped in a clamp, and it therefore only unreliably provides a patent fenestration. If the surgeon really wants a patent fenestration, the heart has to be stopped and the aorta cross-clamped, which adds to the cross-clamp time and imposes a more difficult dissection around the aorta or worse, the Damus root.^3^ So, let us be clear. We are having these endless debates, not for the better sake of the patients, but because it is technically difficult and annoying for the surgeons to do a fenestration during an extra-cardiac conduit and for no other reason. Because if it were technically not challenging, or if we were all doing the lateral tunnel, everybody would do a fenestration.

So, should we stick to the suggested rule of creating a fenestration only if the preoperative pressure is superior to 13 mm Hg? Maybe not so strictly for 2 reasons. First, we are not convinced that 14 mm Hg is so different from 13 or 12 mm Hg. It has been clearly shown that even a very small volume of administration during catheterization can alter these numbers.^4^ Second, we should not believe that the early outcomes after Fontan surgery are essentially dependent on their preoperative characteristics. There is a reason why it has been so difficult to identify factors predictive of early adverse outcomes after Fontan surgery, and this is because one of the biggest factors that impact the outcome is the one that we do not measure: the technical quality of the Fontan construction. We have previously demonstrated the many pitfalls of this surgery that can impact the flow through the Fontan circuit.^5^ And a fenestration will come in handy for all those who have these slight kinks in the Fontan circuit or the pulmonary artery anastomosis.

So, this manuscript by Hill et al^1^ beautifully delineates that if patients have even slightly elevated preoperative pulmonary artery pressures, their Fontan circuit should be fenestrated. We should still encourage surgeons to envision the benefits of a policy. A policy can provide a road map to help achieve benefits for the majority of people, even if some may not benefit from it. It is true that some of us could drive on a highway at very high speeds for long periods without being harmed or harming anybody. But as a policy, it is better for the public to stick to some speed limits. Thus, it might be better to stick to a policy of fenestration for the overall population of patients undergoing the Fontan surgery. And remember, as a surgeon, if you do not want to do it, it is simply because you find it annoying or difficult. There are no other reasons.

## Funding support and author disclosures

The authors have reported that they have no relationships relevant to the contents of this paper to disclose.
